# Preliminary Study on Enzymatic-Based Cleaning of Cation-Exchange Membranes Used in Electrodialysis System in Red Wine Production

**DOI:** 10.3390/membranes9090114

**Published:** 2019-09-03

**Authors:** Myriam Bdiri, Asma Bensghaier, Lobna Chaabane, Anton Kozmai, Lassaad Baklouti, Christian Larchet

**Affiliations:** 1 Institut de Chimie et des Matériaux Paris-Est (ICMPE), Université Paris-Est, UMR 7182 CNRS, 2 Rue Henri Dunant, 94320 Thiais, France; 2 Membrane Institute, Kuban State University, 149 Stavropolskaya Street, Krasnodar 350040, Russia; 3 Department of Chemistry Sciences and Arts College, Al-Rass Province Qassim University, BP35, (KSA) Ar Rass 58876, Saudi Arabia

**Keywords:** ion-exchange membrane, tartaric stabilization of wine, enzymatic cleaning, organic fouling

## Abstract

The use of enzymatic agents as biological solutions for cleaning ion-exchange membranes fouled by organic compounds during electrodialysis (ED) treatments in the food industry could be an interesting alternative to chemical cleanings implemented at an industrial scale. This paper is focused on testing the cleaning efficiency of three enzyme classes (β-glucanase, protease, and polyphenol oxidase) chosen for their specific actions on polysaccharides, proteins, and phenolic compounds, respectively, fouled on a homogeneous cation-exchange membrane (referred CMX-Sb) used for tartaric stabilization of red wine by ED in industry. First, enzymatic cleaning tests were performed using each enzyme solution separately with two different concentrations (0.1 and 1.0 g/L) at different incubation temperatures (30, 35, 40, 45, and 50 °C). The evolution of membrane parameters (electrical conductivity, ion-exchange capacity, and contact angle) was determined to estimate the efficiency of the membrane′s principal action as well as its side activities. Based on these tests, we determined the optimal operating conditions for optimal recovery of the studied characteristics. Then, cleaning with three successive enzyme solutions or the use of two enzymes simultaneously in an enzyme mixture were tested taking into account the optimal conditions of their enzymatic activity (concentration, temperatures, and pH). This study led to significant results, indicating effective external and internal cleaning by the studied enzymes (a recovery of at least 25% of the electrical conductivity, 14% of the ion-exchange capacity, and 12% of the contact angle), and demonstrated the presence of possible enzyme combinations for the enhancement of the global cleaning efficiency or reducing cleaning durations. These results prove, for the first time, the applicability of enzymatic cleanings to membranes, the inertia of their action towards polymer matrix to the extent that the choice of enzymes is specific to the fouling substrates.

## 1. Introduction

Organic Fouling of ion-exchange membranes (IEMs) represents an important issue for industrials and researchers in all fields of membrane and electromembrane systems. This phenomenon concerns the deposition or the adsorption of undesirable organic matters contained in effluents and beverages treated on the surface or inside the membrane matrix. In IEMs the mechanisms involved in fouling are principally caused by electrostatic interactions between fixed functional sites of the polymer, as well as charged and ionisable molecules in solutions [1]. Interactions are also caused by hydrogen bonds between linked water in matrix and heteroatoms (O, N, or S) of organic foulants, and hydrophobic interactions (π–π or n–π stacking) due to the stacking of aromatic rings in both treated solutions and IEM polymers [2,3]. Organic fouling alters the membranes’ physicochemical characteristics by increasing their electrical resistance and changing their surface properties such as hydrophobicity [4,5,6], and requires the implementation of regular cleaning cycles that increase the process cost in industry.

Chemical cleaning procedure is one of the most common and easy practices for the cleaning of IEMs in electrodialysis (ED) and electromembrane processes in general, especially the clean-in-place (CIP) operations that are currently implemented at an industrial scale. CIP generally involves the use of alternating washing sequences by acid and alkali solutions with or without additional surfactants [7], which generates prolonged and repetitive contacts of polymer materials of IEMs with solutions of low and high pH. Alkali cleaning solutions facilitate the removal of organic particles from the surface by the modification of the ionisable charges, making them more soluble and provoking the saponification of the lipids and acid solutions, which are also efficient for the removal of mineral particles [8]. However, recent studies have demonstrated that prolonged contact with acids and bases generates significant mechanical and structural alterations of the membrane polymer matrix marked by the apparition of structure defects as well as significant membrane swelling which lead to a drop in the IEMs selectivity and performance, and reduce their life duration [4,5,9,10,11,12].

It is important to consider the efficiency, processability, and cost of the cleaning methods, as well as the effects of chemical solutions on the membranes’ performance and lifetime. It is consequently essential to develop more gentle cleaning strategies with minimum drawbacks on membrane materials.

The application of biological cleaning solutions that involve the use of enzymatic agents could be an advantageous alternative to the chemical solutions or could at least represent a new method that reduces the frequency of chemical cleanings. Enzymes have a three-dimensional protein structure and react very specifically on their substrate with catalytic properties [13], which makes their action specific to the nature of each foulant. To date, enzymes are not communally used for the cleaning of IEMs, and to our knowledge, no scientific research on the enzymatic cleaning of IEMs has been reported. However, enzymatic procedures have been studied and used at an industrial scale for the cleaning of porous membranes based on polymer materials fouled by organic compounds during filtration processes such as polyethersulfone (PES) ultrafiltration (UF) membranes fouled by proteins [14]. Other studies compared enzymatic cleaning to usual chemical cleaning (alkali/acid) of UF membranes fouled with proteins [15], or investigated the use of enzymes with chemical surfactants in alternating cleaning sequences of polysulfone UF membranes fouled with bovine serum albumin (BSA) and whey [16]. Enzyme activity was found to be targeted, efficient, and without negative effects on membrane materials, and to increase the cleaning efficiency when used prior to surfactant agents. Most studies involving porous membranes′ cleaning with enzymes have been based on the use of hydrolases, a specific class of enzymes that catalyze hydrolysis reactions and allows degradation of organic matter in an aqueous medium. There are several kinds of hydrolases, and the most efficient in the case of organic fouling during beverage treatments or in the dairy industry are the proteases that hydrolyze peptidic bonds in proteins, the glucanases that hydrolyze osidic bonds in polysaccharides, etc. [13,17]. These actions facilitate the solubilization of organic matter or the degradation of complex structures that could accumulate in the pores (in the case of porous membranes) or in the structure defects and interstitial spaces in the case of dense membranes based on polymer matrix [5,10].

If one takes into account the protein nature of an enzyme, its activity could be highly affected, inhibited, or even irreversibly denaturized by the structure changes that could occur under different conditions, such as pH and temperature. The effectiveness of the enzyme’s action also depends on its concentration in the medium in relation to the concentration of the substrates and its chemical structure [13]. Consequently, the use of enzymes in membrane cleaning operations requires prior optimization of the operating conditions in order to improve the washing efficiency [14,16,18]. The use of a moderate pH to comply with the conditions of enzymatic activity is therefore an advantage for ion-exchange membranes, a reason why these kinds of agents could be interesting as an alternative cleaning to the use of chemicals such as acids, bases, detergents, or oxidizing agents.

This work was performed to study the feasibility of enzymatic cleaning of homogeneous CMX-Sb fouled by red wine at advanced stages of tartaric stabilization by ED. Tartaric stabilization is used to reduce the concentration of potassium hydrogen tartrate (KHT) in wine to avoid its precipitation into the bottle and preserve wine quality. Conventional electrodialysis is the most commonly used configuration for this technique, it consists of an electrodialysis cell composed of a succession of chambers. Each ED chamber is formed by a feed compartment (diluate) corresponding to the treated wine, and a brine solution (concentrate) that usually corresponds to a solution of KCl, or KNO_3_, etc., and each compartment is formed by a cation- (CEM) and an anion-exchange membrane (AEM). The ED cell is constituted of a succession of chambers formed by alternating the AEM and CEM [19].

In this study, three enzymes were used for their specific action on fouled organic compounds: Rohalase^®^ BXL (β-glucanase for the hydrolysis of the peptide bonds of proteins), Corolase^®^ 7089 (protease for the hydrolysis of high molecular protein into low molecular peptides), and Tyrosinase^®^ (polyphenol oxidase for the oxidation of phenolic acids and polyphenolic substrates). Cleaning tests were performed on industrially fouled CMX-Sb, and the evolution of physicochemical parameters of the treated membranes was determined to estimate the cleaning efficiency. The principal objectives of the cleaning tests performed on the fouled CMX-Sb were: optimizing the operating conditions for a maximum efficiency for each enzyme, and applying the three enzymatic solutions separately, successively, and simultaneously in an enzyme mixture.

## 2. Materials and Methods

### 2.1. Membranes

Homogeneous cation-exchange membranes CMX-Sb (Astom, Japan) were used in this study, a batch of new membranes (CMX-Sb(n)) and a batch of industrially used membranes (CMX-Sb(u))—taken from an ED stack after ~2738 h of tartaric stabilization of red wine before replacement.

Used membranes are extensively used in industry and are consequently much damaged. It would be interesting to extend their life duration. CMX-Sb are comprised of a poly(styrene-co-divinylbenzene) (PS-DVB) copolymer with sulfonic groups and reinforced by a PVC cloth. As the same batches of CMX-Sb were previously characterized and studied for chemical cleaning or fouling mechanism identification [5,10], the characteristics determined are summarized in Table 1. It involves the ion-exchange capacity (IEC), thickness (*T*_m_), membrane electric conductivity (κ*_m_*), contact angle (θ), water content or water uptake (*W*_C_), the volume faction of the inter-gel solution *f*_2_, and tensile strength parameters: Young′s modulus (*E*) and rupture point (Rp).

In our study, we were mainly interested in following the evolution of the more significant parameters: κ*_m_*, IEC, and θ after enzymatic cleanings. Table 1 shows that after prolonged industrial use, the values of both IEC and κ*_m_* decreased by more than 50% [6,20] under the accumulation of colloidal particles of phenolic compounds that inhibit partially or totally the functional sites. The organic foulants of wine solutions are principally formed of relatively dense low-conducting organic colloidal particles in the inter-gel spaces [21] of the IEM polymer matrices where they replace the electroneutral solution and lead to the decrease in electric conductivity of the fouled membrane [10].

### 2.2. Enzymatic Agents

Three kinds of enzymes were chosen for the cleaning solutions. The choice of enzymes was made according to their properties to degrade the main molecules present in the wine and susceptible to foul the membranes. Rohalase^®^ BXL (pH_opt_ = 4.5, T_storage_ ≤ 10 °C) and Corolase^®^ 7089 (pH_opt_ = 7, T_storage_ = [0–6] °C) were purchased from from AB enzyme (Darmstadt, Germany) in liquid form (~5 g L^−1^).

Tyrosinase^®^ (pH_opt_ = 6.5, T_storage_ = −20 °C) was purchased from Sigma Aldrich (St. Louis, MO, USA) in lyophilized form.

Optimum pH for enzymatic activity is given by the suppliers, and it was recommended to work in a temperature interval varying between 30 and 50 °C. Temperature storage was respected to avoid the loss on enzyme activity.

Rohalase^®^ BXL is a β-glucanase compound with high content of β-1.3- and β-1.6-glucanase which principally perform hydrolysis of the osidic bonds in polysaccharides. The enzyme contains high protease side activities that correspond to the hydrolysis of protein peptide bonds. It was obtained from specific cultures of a classically modified Trichoderma strain. Rohalase^®^ BXL belongs to food and specialties enzymes′ category which is used for winemaking. The enzyme splits colloids and β-glucane derived from botrytis infected grapes and improves the filterability of wines [22].

Corolase^®^ 7089 is a neutral protease that contains exclusively endopeptidase activity responsible for the hydrolysis of high molecular protein into low molecular peptides. An endopeptidase is a form of protease that breaks the bonds inside the protein chain from several sources. It is obtained from Bacillus subtilis cultures. Corolase^®^ 7089 can be used in the fermentation of a variety of alcoholic beverages such as beer, wine, and spirits [23].

Tyrosinase^®^ principal activity corresponds to the catalysis of the mono and diphenolic compounds oxidation to corresponding quinones with the concomitant reduction of molecular oxygen to water. In addition, Tyrosinases oxidize phenolic acids and polyphenolic substrates such as catechins. It is obtained principally from mushrooms and bacteria [24,25]. This kind of tyrosinase has been chosen to act on the phenolic compounds that are abundant in red grapes, which represent the raw material for red wines and can contain on average 3–6 g of total polyphenols per liter [26].

### 2.3. Chemicals

Glacial acetic acid (>99.85%), sodium acetate (99 atom% ^13^C), potassium phosphate dibasic, potassium phosphate monobasic for buffer solutions, and sodium hydroxide (anhydrous; >98%) were purchased from Sigma Aldrich (St. Louis, MO, USA). Hydrochloric acid 37% was purchased from VWR Chemicals (France) and crystalline powder of sodium hydroxide (>99%) from Alfa Aesar (Germany).

### 2.4. Protocols

New CMX-Sb were stabilized before characterizations and cleaning treatments according to the protocol described in the French standard NF X 45-200 [27] and used CMX-Sb were directly stored in 0.1 M NaCl conditioning solution to avoid the possible evolutions of these membranes.

First, for the optimization of enzyme concentrations, cleaning tests were performed for each enzyme with two different concentrations taken into account the masse of pure enzymes per liter of solvent (0.1 and 1.0 g L^−1^) at five distinct incubation temperatures (30, 35, 40, 45, and 50 °C). The choice of preconized concentrations was based on two principal information: one given by our enzyme supplier and the other taken from our bibliographic study. The enzyme supplier (AB enzymes) gave a recommended concentrations of 0.5 g L^−1^ for Rohalase^®^ and 0.2 g L^−1^ for Corolase^®^ during at least 2 h for the cleaning of cross-membranes and filter cartridges. Usual concentrations used in previous works for the enzymatic cleaning of porous membranes are 0.1 [14,17] to 0.75 wt % [16].

The solvent of each enzyme corresponded to a specific buffer that maintains its pH for optimal activity (sodium acetate at pH 4.5 for Rohalase^®^, and potassium phosphate at pH 6.5 and 7 for Corolase^®^ and Tyrosinase^®^, respectively). For each enzyme, 6 samples of 5 cm^2^ were cut from the used CMX-Sb, and each sample was soaked in 10 mL of the diluted enzyme solution at specific concentrations (0.1 or 1.0 g L^−1^) and temperatures (30, 35, 40, 45, or 50 °C) for 20 h in 50 mL closed flasks. Temperatures were maintained by soaking the flasks in a thermo-regulated bath. The duration of cleaning was chosen to be ten times longer than that recommended by AB enzymes for cleaning cross-membranes and filter cartridges, since CEMs have a high degree of use and are highly fouled.

After choosing an optimal concentration and incubation temperature for each enzyme, a membrane sample of 5 cm^2^ was soaked in a 10 mL solution of each enzyme at its optimal pH successively during a 10 h period. The duration was half that implemented for each individual enzyme because the different cleaning modes were combined and to reduce the total time of cleaning from 60 to 30 h for each membrane. Before changing the solution, the sample was rinsed with 100 mL ultra-pure water (Milli-Q water).

To finish, a cleaning operation was performed on a 5 cm^2^ membrane sample using a 10 mL mixture of two enzymes, Corolase^®^ and Tyrosinase^®^ because of their close values of optimal pH in a potassium phosphate buffer solution at 6.8 pH for 10 h at their optimal concentration and temperature.

Following all the cleaning operations, the IEC, κ*_m_*, and θ were determined to estimate the efficiency of the membranes’ parameter recovery.

### 2.5. Analysis

#### 2.5.1. Ion-Exchange Capacity

The ion-exchange capacity (IEC) was determined by soaking the CMX-Sb sample in 25 mL cm^−2^ of 1 M HCl for 2 h, and then rinsing it with ultra-pure water (Milli-Q water). The sample was then immersed in a solution at 23 mL cm^−2^ of 0.1 M NaCl + 2 mL cm^−2^ of 0.1 M NaOH for at least 2 h. The hydroxide amount was immediately determined by titrating using a 0.01 M HCl. For CMX-Sb(u), the immersion times in the corresponding solutions were doubled to ensure the internal equilibrium of the fouled membranes. Ultimately, the dry mass was determined using a moisture analyzer HB43-S Mettler-Toledo in 40 min at 80 °C.

#### 2.5.2. Electric Conductivity

Membrane electric conductivity (κ*_m_*) was measured in a 1 M NaCl solution using a clip-type cell and a conductivity meter CDM92 (Radiometer) at 25 ± 0.5 °C maintained in a thermo-regulated water reservoir. Two measurements of the NaCl solution′s electric resistance were needed, with (R_2_) and without (R_1_) the membrane. Thus, we used the following equation:

$$\kappa_{m}=\frac{T_{m}}{\left(R_{2}-R_{1}\right)\;A}$$

where A is the area of the electrodes (1 cm²).

#### 2.5.3. Contact Angle

An FM40 Easy Drop (KRUSS, Germany) was used to determine the contact angle (θ) between a pure water drop and the membrane surface lightly wiped with a filter paper after rinsing with ultra-pure water (Milli-Q water).

## 3. Results and Discussion

### 3.1. Optimizing the Operating Conditions

**Figure 1:**
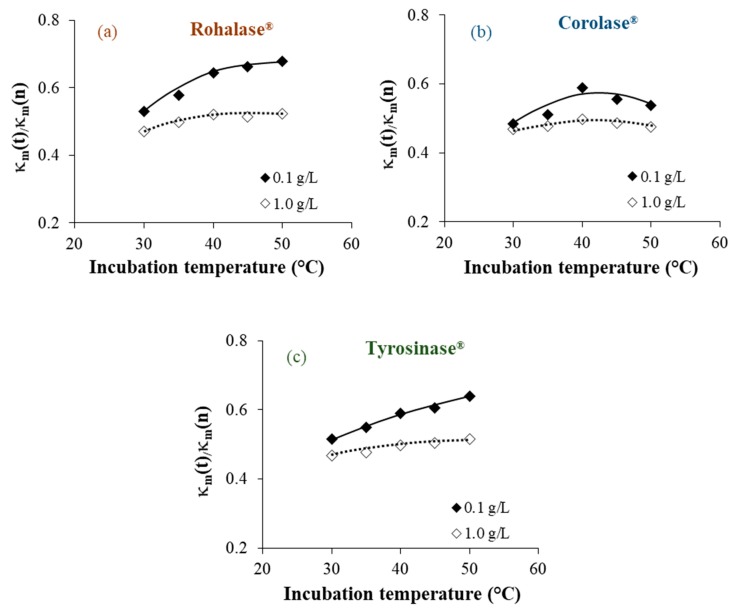
Dependence of κ*_m_*(t) for the treated CMX-Sb on incubation temperatures and concentrations of Rohalase^®^ (**a**); Corolase^®^ (**b**), and Tyrosinase^®^ (**c**).

**Figure 2:**
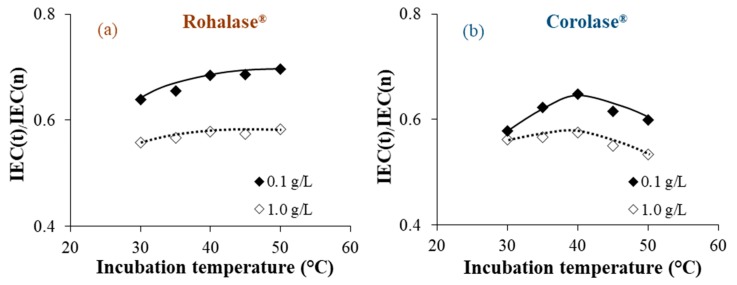

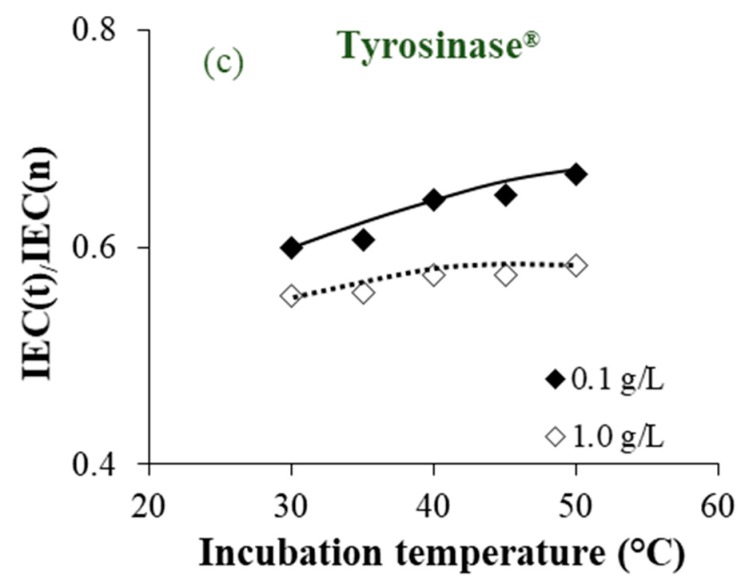
Dependence of the IEC(t) for the treated CMX-Sb on incubation temperatures and concentrations of Rohalase^®^ (**a**); Corolase^®^ (**b**), and Tyrosinase^®^ (**c**).

**Figure 3:**
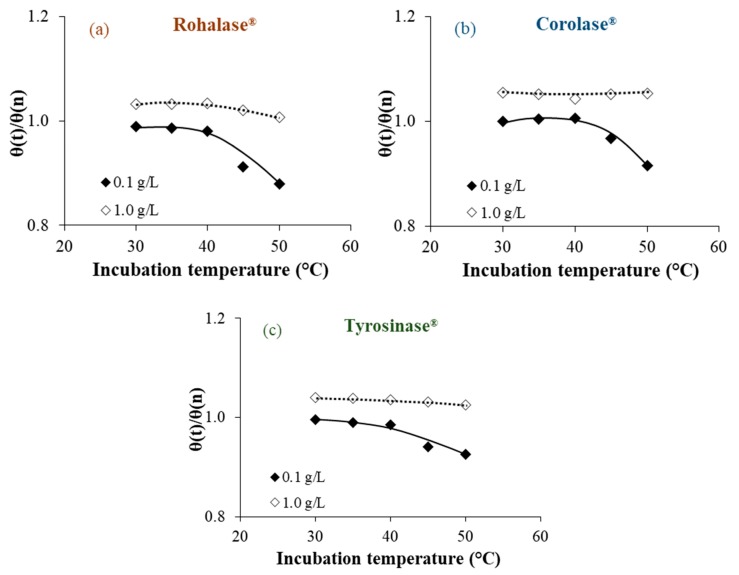
Dependence of the θ(t) for the treated CMX-Sb on incubation temperatures and concentrations of Rohalase^®^ (**a**), Corolase^®^ (**b**), and Tyrosinase^®^ (**c**).

In order to highlight the evolution of the studied parameters following the 20 h cleaning operations with each enzyme solution at different concentrations (0.1 and 1.0 g L^−1^) and temperatures (30, 35, 40, 45, and 50 °C), and compared to the initial values for CMX-Sb(n), evolution of the κ*_m_*(t)/κ*_m_*(n), IEC(t)/IEC(n), and θ(t)/θ(n) ratios are reported in Figure 1, Figure 2 and Figure 3, respectively. Since the studies CEMs are not perfectly homogeneous due to their method of preparation and the heterogenic distribution of the organic fouling on their whole surface, their parameters could sensitively change from one sample to another. This is due to the fact that the ratios to the initial values were taken into account.

We observed that after 20 h of cleaning, the best results were obtained at lower enzyme concentrations (0.1 g L^−1^) and at a common temperature of 50 °C. As enzymes already have catalytic activity at higher concentrations (1.0 g L^−1^), it could be possible that enzymes are proteins that act as foulants. This hypothesis was suggested by Muñoz-Aguado et al. [16], where the same enzymatic cleaning was successively repeated during alternating enzyme-surfactant cleaning sequences on UF membranes fouled with BSA and whey.

Under these conditions, significant percentages of the studied parameters were recovered after the cleaning operations compared to the non-treated membranes. These were the percentages obtained after cleaning with each enzyme solution: Rohalase^®^ (+45% in κ*_m_*, +23% in IEC and −16% of the θ), Corolase^®^ (+ 26% in κ*_m_*, + 14% in IEC and −13% of the θ), and Tyrosinase^®^ (+37% in κ*_m_*, +18% in IEC and −12% of the θ). The increase observed for the membranes’ electric conductivity and the ion-exchange capacity following the cleaning procedures could be attributed to the efficiency of internal cleaning. Our previous studies, on the same CMX-Sb and their homologue anion-exchange membranes (AMX-Sb), show that internal organic fouling after prolonged use leads to partial inhibition of fixed functional sites due to electrostatic interactions with charged groups and to the accumulation of relatively dense low-conducting organic colloidal particles in the inter-gel spaces where they replace the electroneutral solution [5,10]. Despite the three-dimensional protein structures of enzymes, their relatively high molecular weight [13], and the dense structure of the polymer material of the CMX-Sb tested, the enzymes acted upon their specific substrates as shown in Figure 1, Figure 2 and Figure 3, which confirm that the enzymes penetrated into the polymer matrix. It could be explained by the high degree of swelling of the CMX-Sb(u) (an increase by 50% in thickness and water content was observed for CMX-Sb(u) comparing to CMX-Sb(n) after prolonged industrial use as shown in Table 1) facilitated the penetration of molecules with high molecular weights such as polysaccharides and proteins during beverage treatments.

The decrease in contact angle indicated a decrease in the hydrophobicity of the membrane′s surface after treatments due to the partial removal of hydrophobic organic foulants.

The membrane’s thickness (*T*_m_) and the tensile strength parameters (*E* and *R*_p_) of the CMX-Sb were also determined after the different enzymatic cleanings, and no significant changes were noticed. It can be concluded that the activity of the tested enzymes had no negative effects on the mechanical properties of the membranes and that it did not provoke additional swelling of the polymer matrix. Consequently, enzymatic agents proved their efficiency for both external and internal cleaning of the studied CMX-Sb. It should be noted that the chosen enzymes are inert towards membrane matrices as their action is specific to their substrates.

Rohalase^®^ principal activity as β-glucanase enzymes is the hydrolysis of polysaccharides. Furthermore, its activity was the most significant according to the higher recovery percentages observed. Polysaccharides, such as arabinanes, galactanes, arabino–galactanes, etc., with a molecular weight ranging from 40 to 250 kDa or β-glucanes that are linear molecules with molecular weight ranging from 25 and 270 kDa, are abundant raw materials for beverage preparation such as fruits (grape, apple, etc.) [28,29,30].

Tyrosinase^®^ principal activity is to catalyze the oxidation of mono- and di-phenolic compounds to corresponding quinones with the concomitant reduction of molecular oxygen to water. One of the more studied side activities of bacterial Tyrosinase^®^ is the oxidation of phenolic compounds such as phenolic acids (caffeic acid) and polyphenolic substrates such as catechins [25,31,32]. Phenolic compounds are amphipathic molecules that contain hydrophobic aromatic rings and hydrophilic phenolic hydroxyl groups [26,33]. They are also abundant in beverages (wines especially red wine, fruits and vegetables juices, and derivatives) and reach more than 6 g of total polyphenols per liter of solution. The amount of such foulant compounds vary from a kind of beverage to another but they are highly responsible for the organic fouling of IEM polymer materials even at low concentrations and after a short duration of contact with solutions. It was demonstrated by Sarapulova and al. [3] that during the first hours of contact with red wine (during an operation of fouling simulation on an anion-exchange membrane), strongly hydrated and relatively small organic molecules, such as anthocyanins, penetrate easily into the matrix. The latest components are colored pigments of general structure in C_15_ (C_6_–C_3_–C_6_) and approximate molecular weight of 200 g mol^−1^ (corresponding to 0.2 kDa) that belong to the flavonoids class, a category of phenolic compounds [34,35]. Bigger molecules such as tannins and proteins can enter the membrane due to matrix swelling and the apparition of structure defects (mainly caused by alkali and acidic cleaning operations in industry [5,9,10]) [3]. This interpretation is also valid in the case of CMX-Sb. In fact, due to the nature of functional sites and their negative charge (sulfonic groups), the phenomenon of Donnan exclusion of hydroxyl groups occurs, which decreases the pH value of the internal charged solution in the membrane compared to the externally treated solution that has an acidic pH generally around 3.5 [1,3,36]. Internal CMX-Sb pH in this condition is slightly more acidic, which favors the presence of positively charged anthocyanins. These anthocyanins are rich in chromene cycles that exist in the form of positively charged flavylium cations and could have more tendency to interact with negatively charged functional groups of the matrix. These cycles are also present in condensed tannins (general structure of tannins in (C_15_)) and contribute to fouling [3,37]. Anthocyanins have a great tendency to interact with simple sugars and polysaccharides, or with homologue species such as other anthocyanins or phenolic acids, and be present in condensed forms or in aggregates [26,38]. Consequently, the action of polysaccharide hydrolysis and phenolic compound oxidation could be useful for the decomposition of such complex structures inside the membranes.

The cleaning strategy using the Corolase^®^ enzyme resulted in less recovery compared to the other two enzymes. It is a protease that hydrolyzes proteins and reduces their structure. Even if the amount of proteins is less important than polysaccharides, simple sugars, and phenolic compounds in food beverages, the efficiency of protease proved the effective hydrolysis of proteins. Indeed, proteins are present in wines at a content of ~1.0 g L^−1^ and could be accumulated in swollen polymer matrices of IEMs in simple or condensed forms with tannins [26]. Rohalase^®^ and Tyrosinase^®^ enzymes have a side protease activity, another reason why the results obtained for these two enzymes are more important.

It might be possible to test enzymatic agents as regular cleaning solutions from the beginning of ED treatments during fouling simulation on ED cells in laboratories. It could be predicted that enzyme activity would be more efficient insofar as it could prevent the accumulation of organic foulants and their side reactions such as condensation, polymerization, etc. inside the membranes’ matrix, and cleaning would be limited to the surface as membranes would not be subjected to the important swelling phenomenon at the first stage of fouling by organic matters.

### 3.2. Membrane Cleaning Using Three Enzymatic Solutions Successively and a Mixture of Two Compatible Enzymes

The previous optimization of enzyme concentrations and incubation temperatures determined the optimal operating conditions for each enzyme: 0.1 g L^−1^ at 50 °C for Rohalase^®^, Corolase^®^, and Tyrosinase^®^. After 30 h of successive cleaning (first Rohalase^®^, second Corolase^®^, and then Tyrosinase^®^) of a used CMX-Sb sample, a recovery of ~50% in κ*_m_*, 33% in IEC, and 15% in θ was obtained. It should be taken into account that the duration of cleaning with each enzyme solution was two times less than the tests during operating conditions′ optimization. It was reduced to 10 h by each enzyme instead of 20 h. It could be assumed that Rohalase^®^ enzymes were efficient for polysaccharides hydrolysis, followed by proteins, and then effective oxidation of phenolic compounds by the catalytic effect of Tyrosinase^®^. This order led to a reduction in the size of foulants’ complex structures such as tannin–polysaccharide aggregates [33] or the condensed forms of anthocyanins–polysaccharides under the principal action of Rohalase^®^. Then Tyrosinase^®^ was able to oxidize their free states.

The order of sample soaking in the enzyme solutions was chosen arbitrarily. For our future studies on IEMs cleaning with enzymatic agents, the effect of the order will be studied to optimize the results obtained and to increase the efficiency of the cleaning sequences.

The mixture of enzymes (enzymes cocktail) for the last cleaning test contained both Corolase^®^ (pH_opt_ = 7) and Tyrosinase^®^ (pH_opt_ = 6.5) because of their close values of optimal pH in a potassium phosphate buffer solution at 6.8 pH. The considered concentration was 0.1 g L^−1^ for each enzyme at an incubation temperature of 50 °C and the treatment duration was chosen to be 10 h. This strategy led to 25% increase in κ*_m_*, 18% increase in IEC, and 13% increase in θ. These results could give an idea about the important effect of Rohalase^®^ for intern cleaning and the reduction of polysaccharides and their condensed forms with phenolic compounds (anthocyanins and tannins) on the CMX-Sb fouling.

Table 2 includes a summary of the recovery rates of the significant IEM parameters studied (κ*_m_*, IEC, and θ) after the different enzymatic cleaning operations.

## 4. Conclusions

This preliminary study includes the enzymatic cleaning of CMX-Sb used for the treatment of red wine by ED in the food industry. Three different enzymes were used for the experiments; Rohalase^®^ BX-BXL (a β-glucanase for the hydrolysis of osidic bonds in polysaccharide), Corolase^®^ 7089 (a neutral protease for the hydrolyses of high molecular protein into low molecular peptides), and Tyrosinase^®^ that principally catalyze the mono- and di-phenolic compounds′ oxidation to corresponding quinones, and also the oxidation of phenolic acids and polyphenolic substrates.

The enzymes′ activities were found to be more efficient at lower concentrations (0.1 g L^−1^). It was assumed that at higher concentrations (1.0 g L^−1^), the enzymes that are basically proteins could foul the polymer matrix.

We identify suitable enzymes for IEM cleaning through this study. However, it would be preferable to choose more specific enzymes taking into account the exact nature of foulants with an adequate evaluation of enzyme activity during the cleaning processes. For ED treatments of red wines, IEMs are in prolonged contact with phenolic compounds, proteins, polysaccharides, and simples sugars, etc. It seems that the best strategy for estimating the cleaning efficiency of enzymatic agents is to study the activity of each enzyme towards its substrate separately on fouled IEM samples after fouling simulations by each of the main foulants. Consequently, the amount of recovered foulants in the cleaning solution could be analyzed (by UV-vis detection, for example) without any interference with other components.

It will be also interesting to investigate enzymatic cleaning on anion-exchange membranes for comparative analysis.

## Figures and Tables

**Table 1:** Summary of the main characteristics of studied CMX-Sb.

Characteristics	CMX-Sb(n)	CMX-Sb(u)
**IEC (mmol g^−1^)**	2.47 ± 0.13	1.40 ± 0.06
***T*_m_ (µm)**	177 ± 3	270 ± 4
**κ** ***_m_* (mS cm^−1^)**	13.4 ± 0.6	6.2 ± 0.4
**θ (°)**	47 ± 1.3	49.3 ± 0.9
***W*_C_ (%)**	26.1 ± 0.9	40.0 ± 2.1
***f*_2_**	0.11 ± 0.01	0.04 ± 0.01
***E* (MPa)**	555 ± 11	264 ± 17
***R*_p_ (MPa)**	29 ± 3	11 ± 1

IEC: Ion-Exchange Capacity. κ_m_: Electrical conductivity. θ: Contact angle. Wc: Water content. *f*_2_: Volume fraction of the inter-gel solution. E: Young′s Modulus. / *R*_p_: Rupture point.

**Table 2:** Summary of the recovery rates of κ*_m_*, IEC, and θ after the different enzymatic cleanings.

Cleaning Operations	Total Duration (h)	% κ*_m_* (mS cm^−1^)	% IEC (mmol g^−1^)	% θ (°)
Rohalase^®^ BXL	20	+45	+23	−16
Corolase^®^ 7089	20	+26	+14	−13
Tyrosinase^®^	20	+37	+18	−12
Successive cleanings	30 (10 h/enzyme)	+50	+33	−15
Enzymes cocktail	10	+25	+18	−13
